# Supplementary figures and images for: scMagnifier: Resolving fine-grained cell subtypes via GRN-informed perturbations and consensus clustering
Source: PLoS Comput Biol. 2026 Jun 18;22(6):e1014167. doi: 10.1371/journal.pcbi.1014167 (PMC13293510; doi:10.1371/journal.pcbi.1014167)

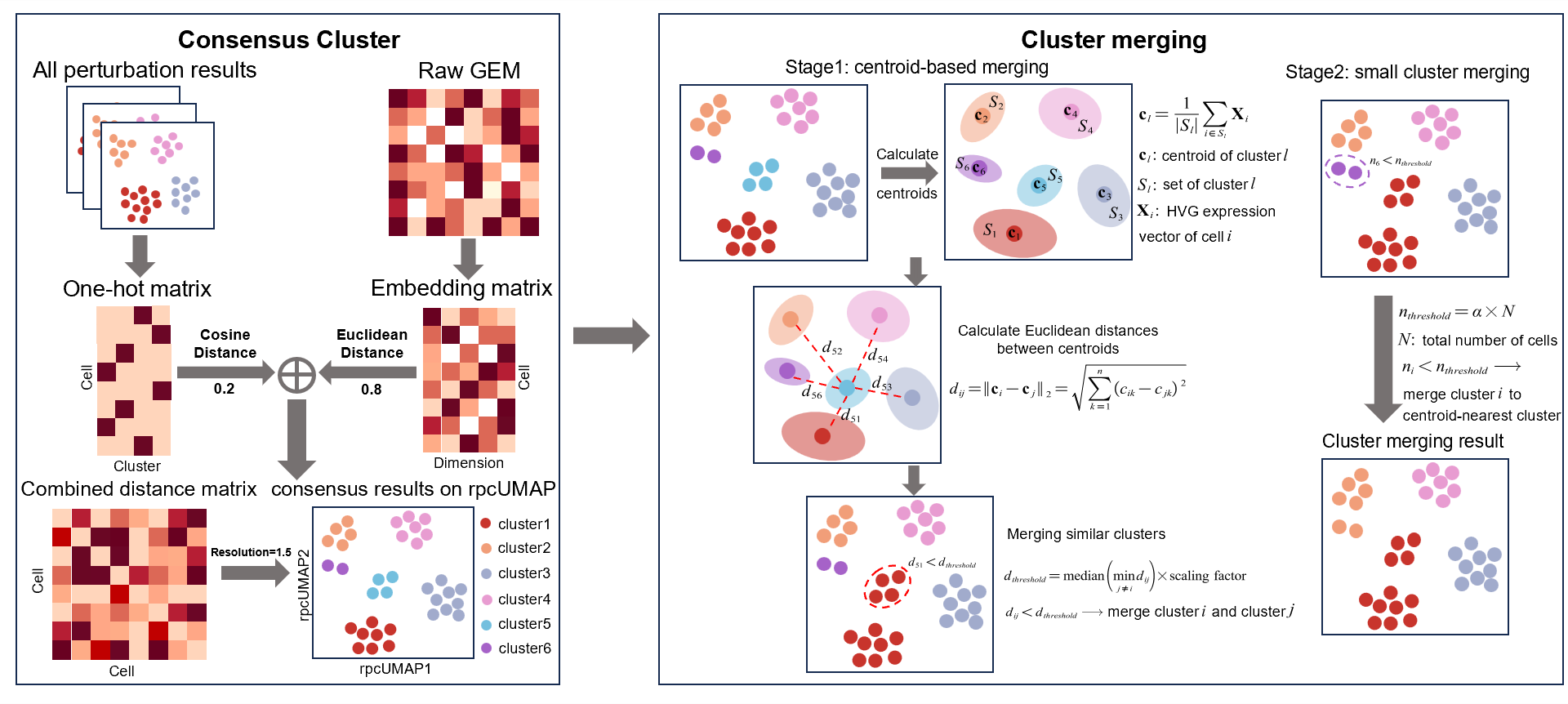

Supplement: S1 Fig — (TIF) [file pcbi.1014167.s001.tif]

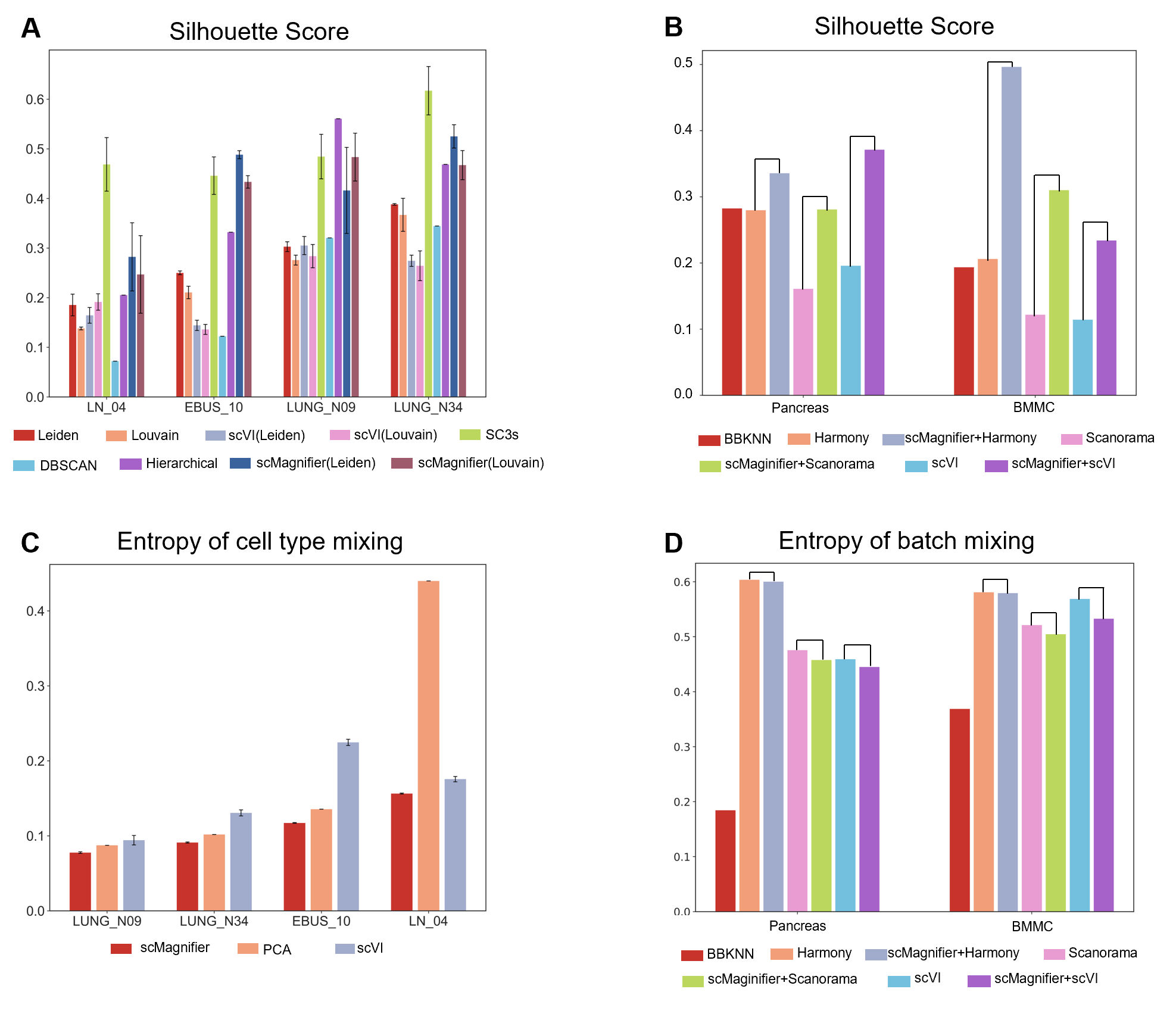

Supplement: S2 Fig — (A) Bar plots illustrating the performance of nine algorithms on four single-batch datasets, evaluated using the silhouette score. (B) Bar plots illustrating the silhouette score for seven batch-correction methods applied alone or in combination with scMagnifier, evaluated on two multi-batch datasets. (C) Cell type mixing entropy of embeddings derived from scMagnifier, PCA, and scVI, evaluated on four single-batch datasets. Entropy of cell type mixing was computed based on neighbors in the corresponding latent space for each method: PCA-based neighbors for PCA, scVI-based neighbors for scVI, and mixed-distance neighbors for scMagnifier. (D) Bar plots depicting batch mixing entropy for seven batch-correction approaches applied alone or integrated with scMagnifier, assessed on two multi-batch datasets. (TIF) [file pcbi.1014167.s002.tif]

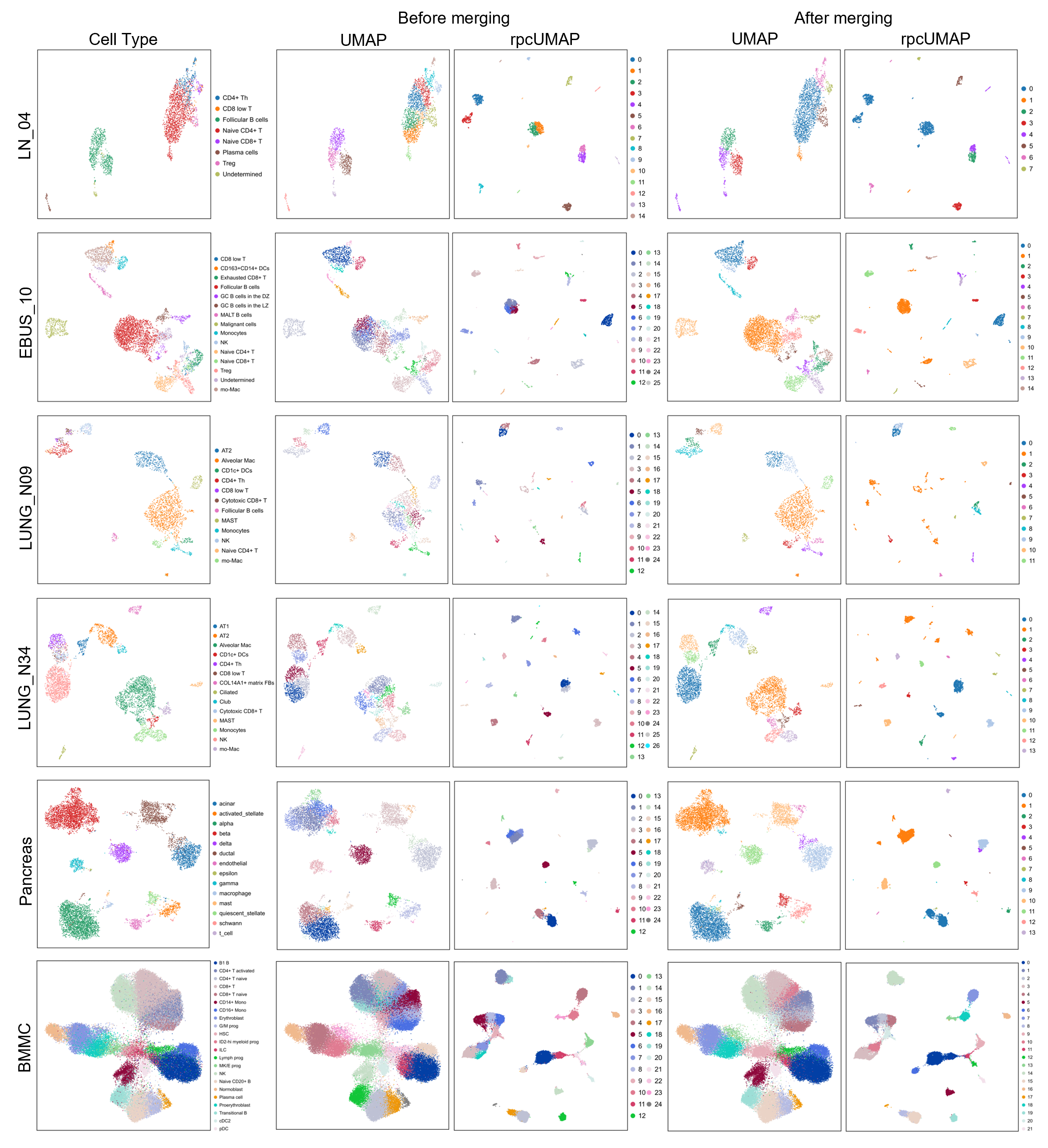

Supplement: S3 Fig — (TIF) [file pcbi.1014167.s003.tif]

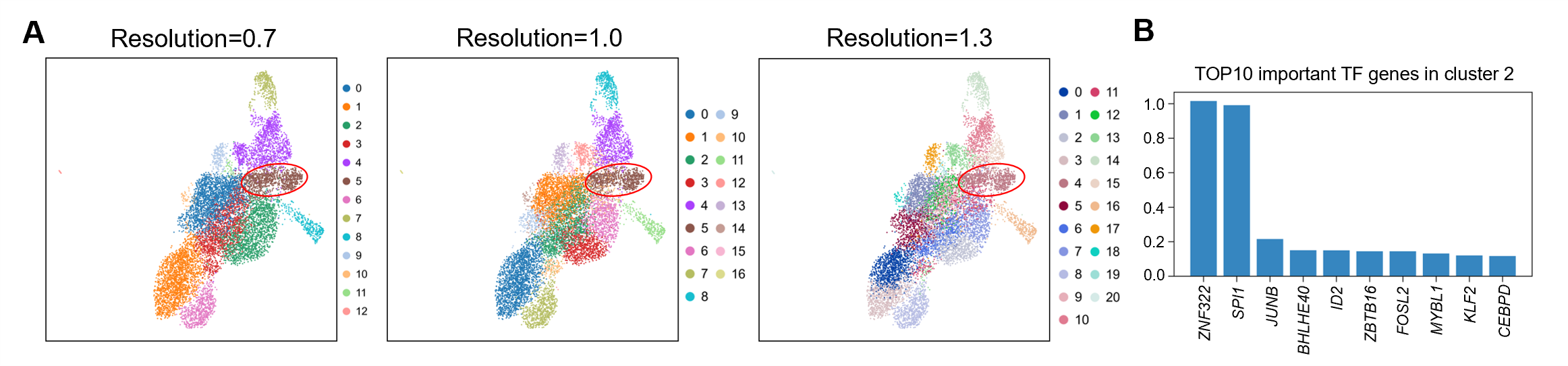

Supplement: S4 Fig — (A) UMAP visualization of clustering results via the Leiden clustering algorithm with increasing resolution parameters (resolution = 0.7, 1.0, 1.3), highlighting MAIT cells and a Th1/Th17-MAIT mixed population were grouped into a single cluster. (B) Bar plot of the top 10 TF genes ranked by importance scores in cluster 2. (TIF) [file pcbi.1014167.s004.tif]

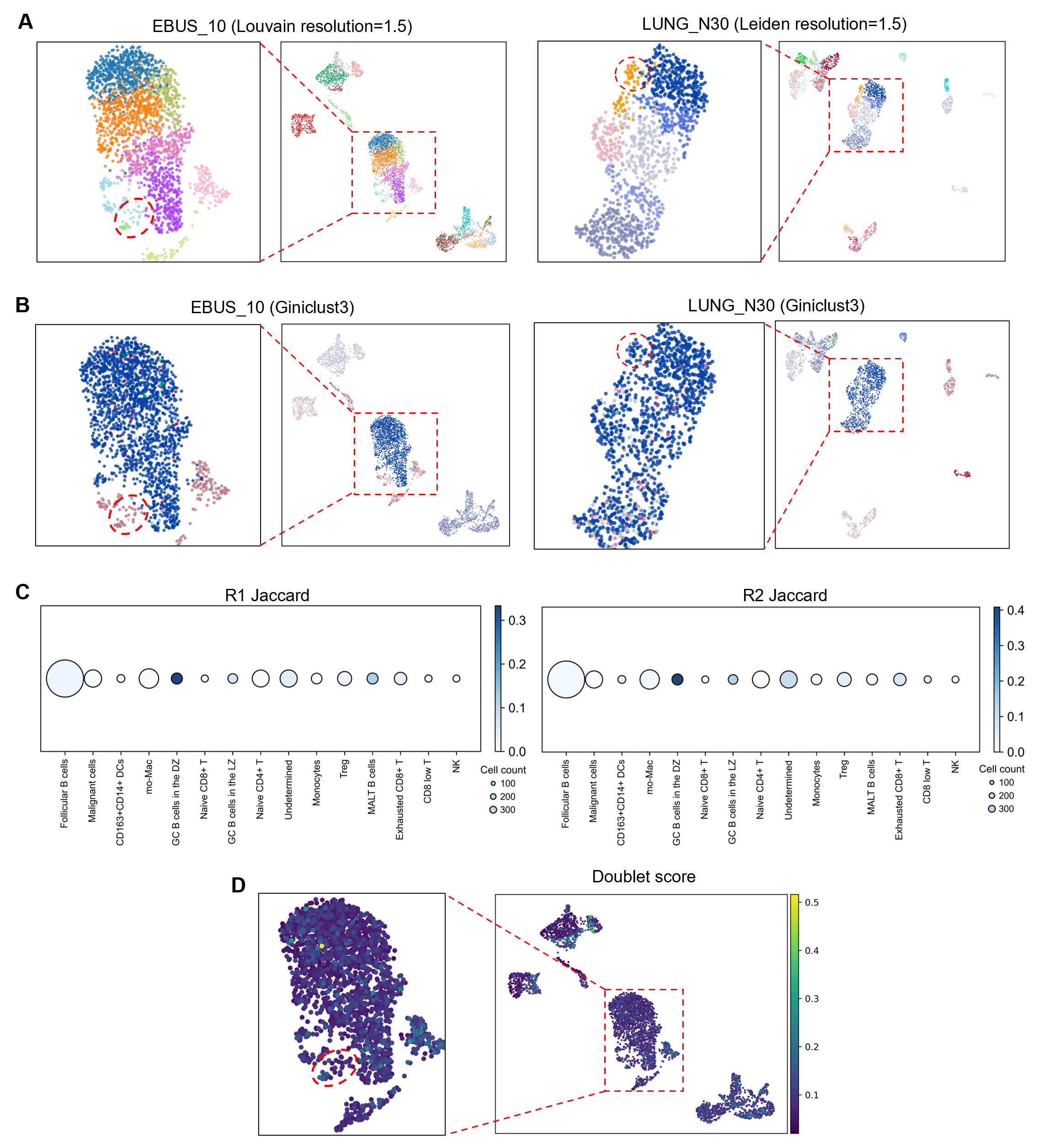

Supplement: S5 Fig — (A) UMAP visualization of clustering results obtained via standard clustering method at high resolution (resolution = 1.5) in EBUS_10 and LUNG_N30 datasets, where rare cell populations fail to be identified as individual clusters. (B) UMAP visualization of rare cell identification results by GiniClust3. (C) Bubble plot showing Jaccard coefficient values between R1/R2 and clusters of the remaining cells grouped by cell-type annotations. (D) UMAP visualization of doublet detection results in the EBUS_10 dataset. (TIF) [file pcbi.1014167.s005.tif]

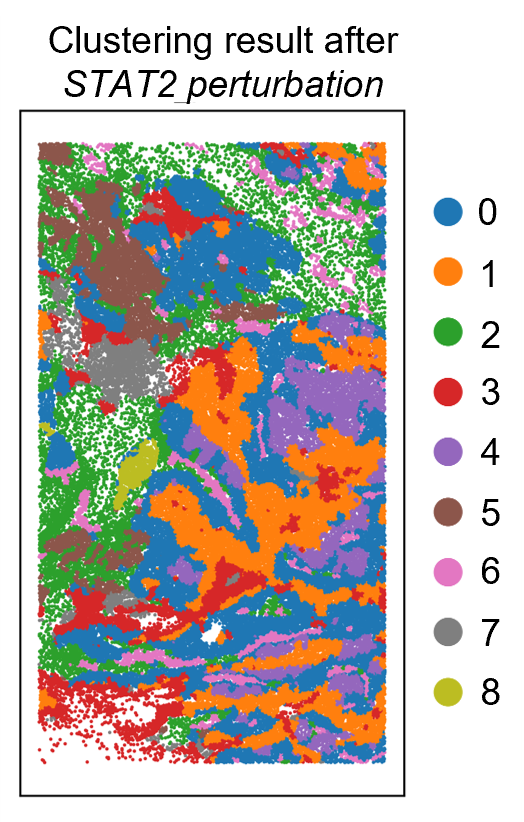

Supplement: S6 Fig — (TIF) [file pcbi.1014167.s006.tif]

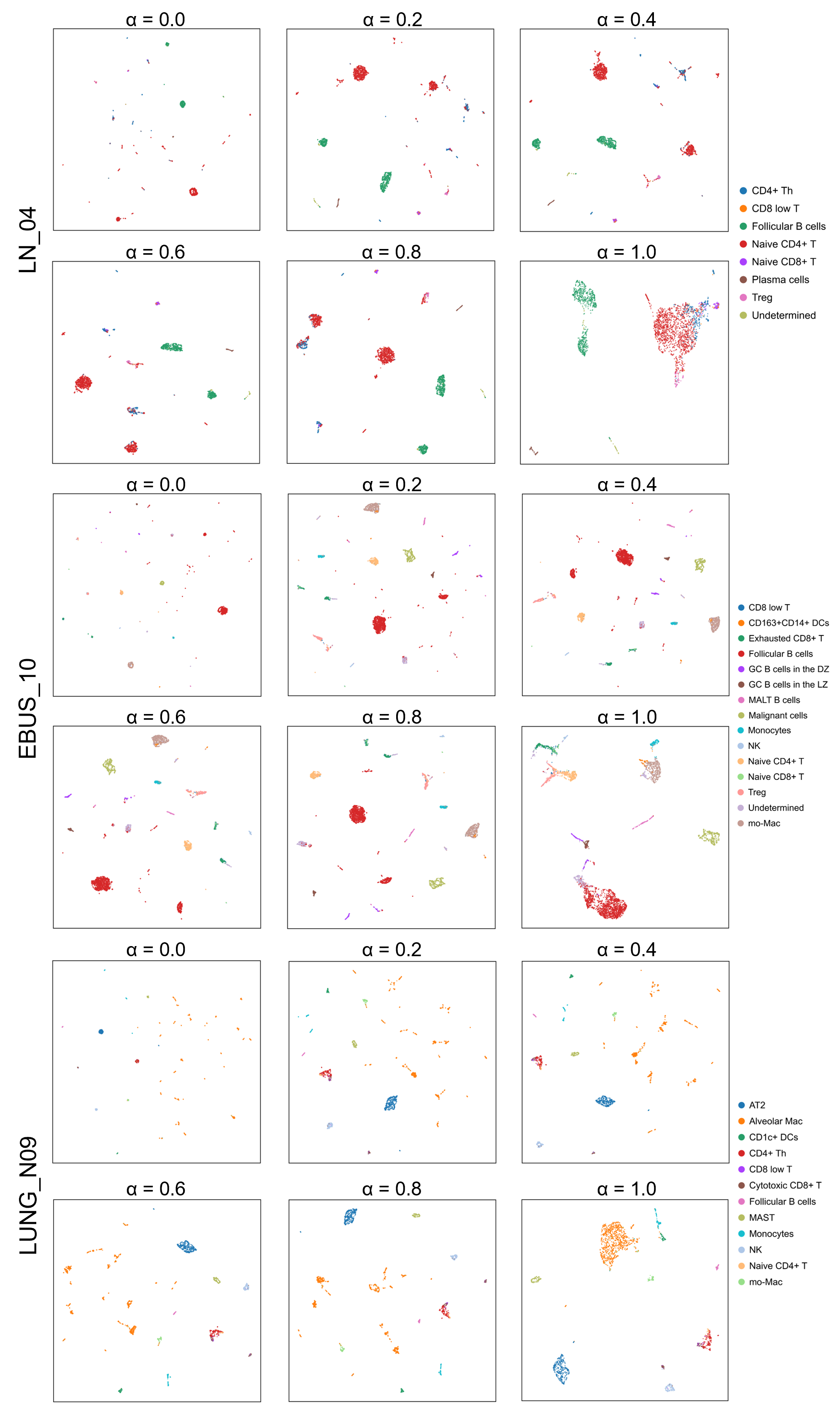

Supplement: S7 Fig — As the α parameter decreases gradually, more small clusters emerge in rpcUMAP visualizations, resulting in an excessively fragmented clustering profile. Thus, we selected α = 0.8 as the default setting, which not only enables the separation of truly distinct clusters by incorporating perturbation-derived information but also avoids over-separation and the generation of numerous boundary small clusters that interfere with result interpretation. (TIF) [file pcbi.1014167.s007.tif]

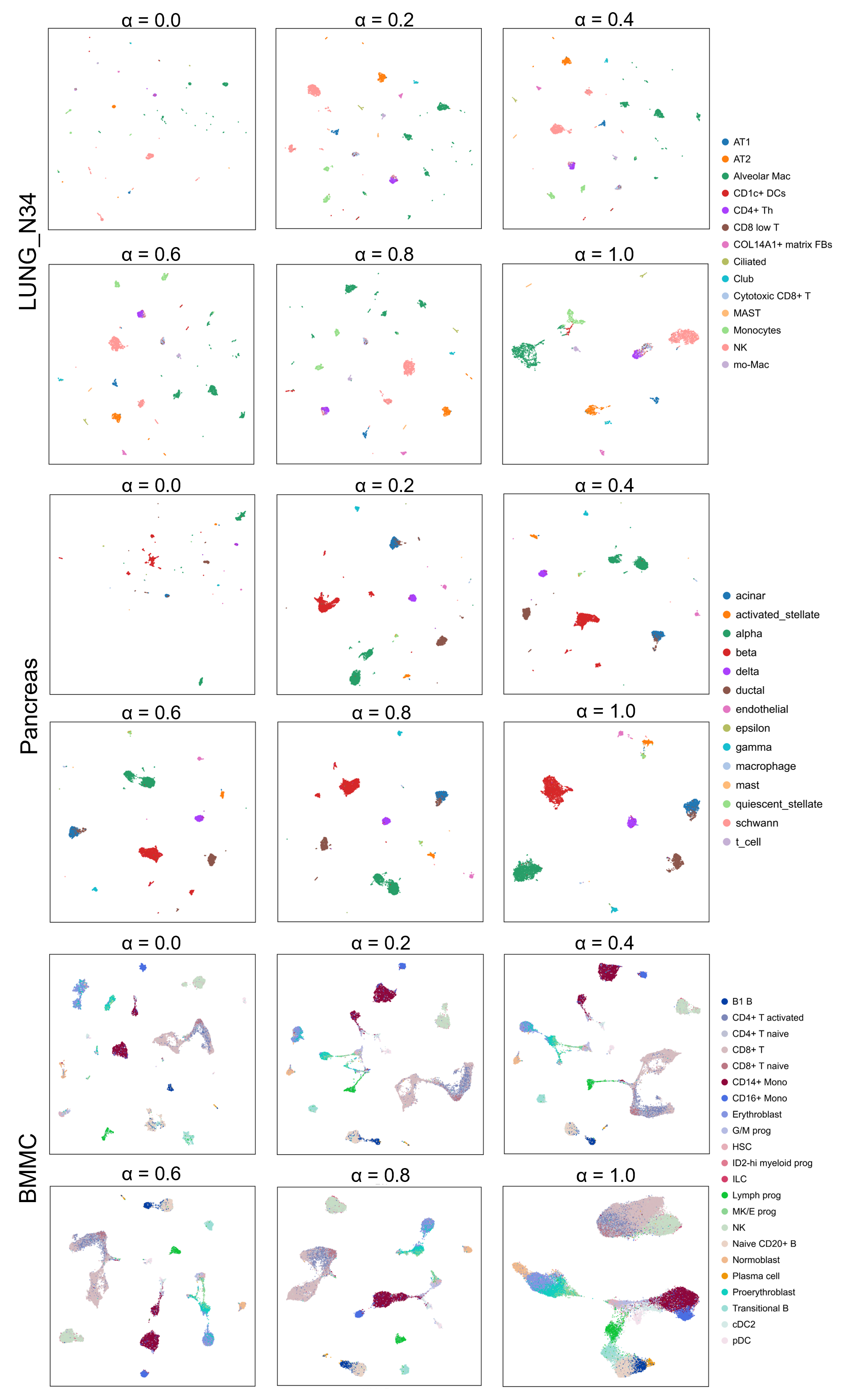

Supplement: S8 Fig — (TIF) [file pcbi.1014167.s008.tif]

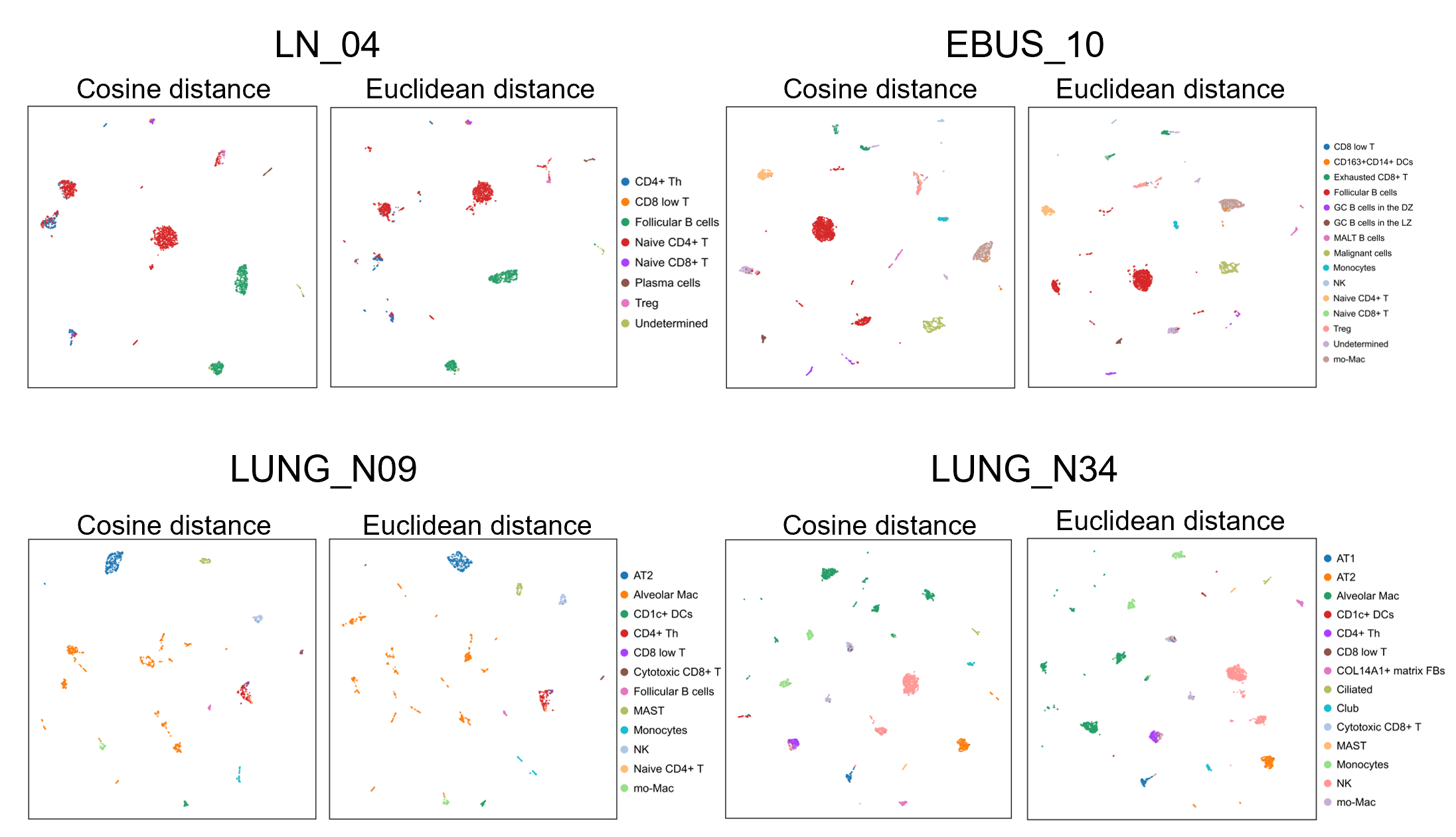

Supplement: S9 Fig — The visualization results derived from cosine distance are less fragmented than those from Euclidean distance, thus cosine distance was selected as the default setting. (TIF) [file pcbi.1014167.s009.tif]

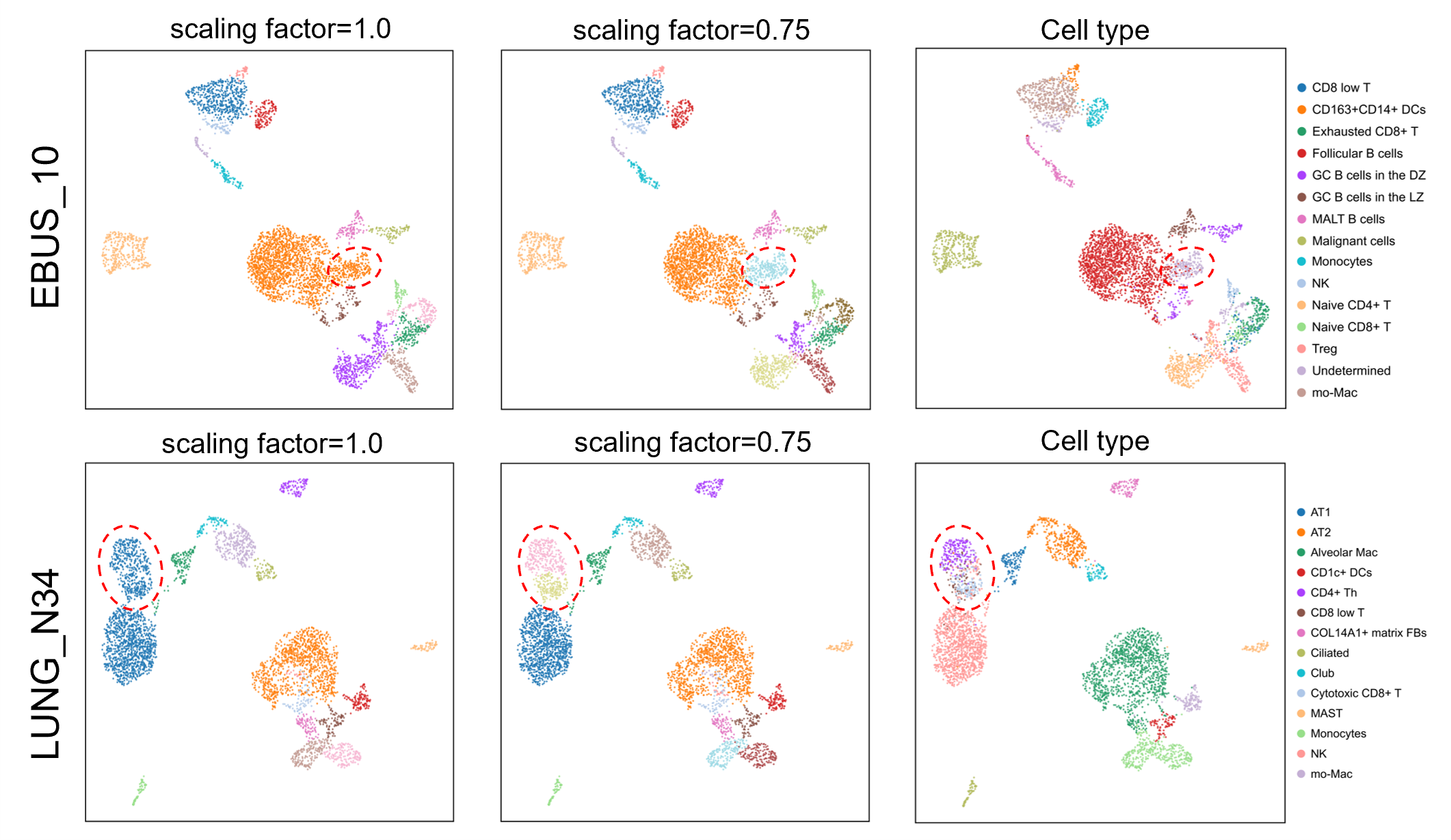

Supplement: S10 Fig — When the scaling factor = 1.0, clusters are over-merged, with different cell type clusters combined into one. Thus, 0.75 was selected as the default value to balance merging efficacy and cluster specificity. (TIF) [file pcbi.1014167.s010.tif]

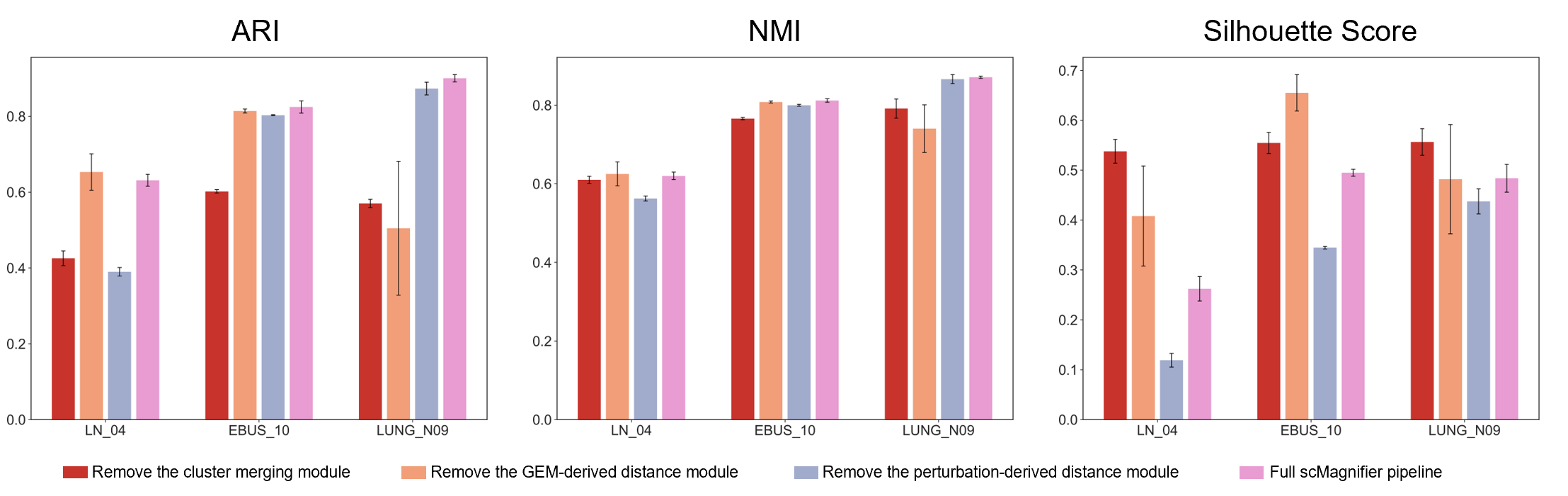

Supplement: S11 Fig — (TIF) [file pcbi.1014167.s011.tif]

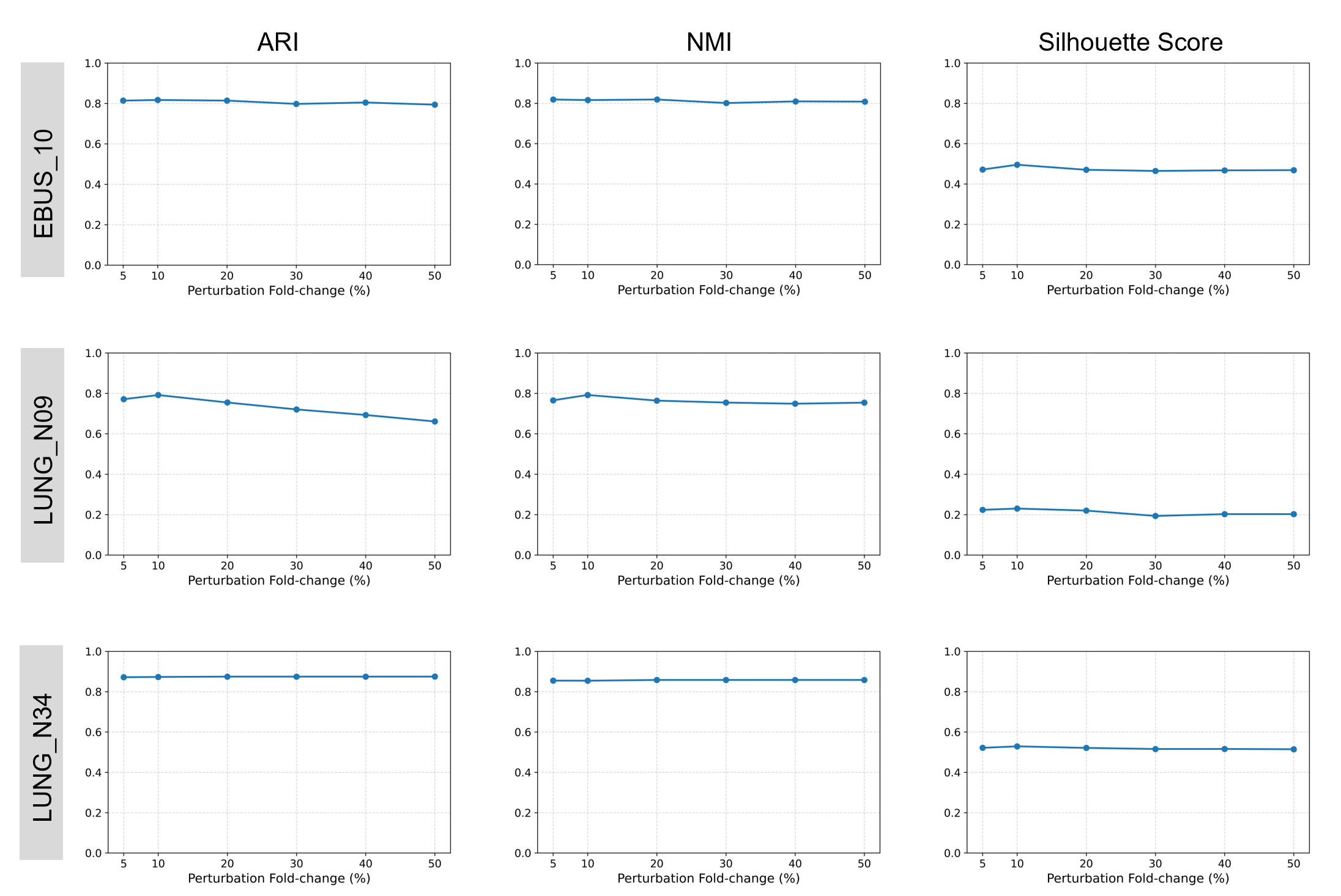

Supplement: S12 Fig — (TIF) [file pcbi.1014167.s012.tif]

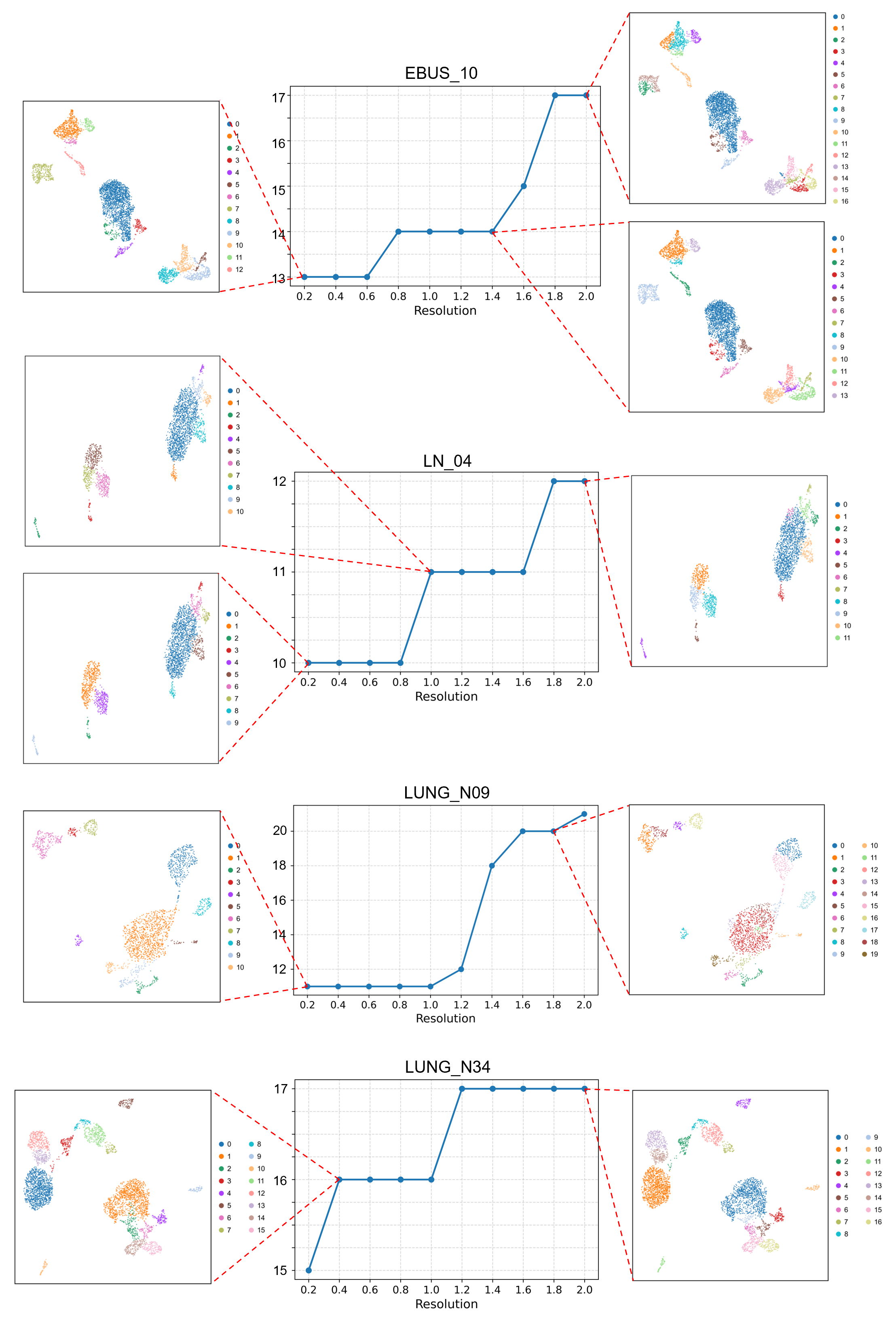

Supplement: S13 Fig — Plateaus indicate stable cluster numbers, with corresponding UMAPs confirming biologically meaningful structure. The resolution parameter in the consensus clustering step was varied from 0.2 to 2.0 with increments of 0.2 (all other parameters were kept at their default values), and the number of detected clusters was plotted as a function of resolution. (TIF) [file pcbi.1014167.s013.tif]
