# Supplementary material for: scMagnifier: Resolving fine-grained cell subtypes via GRN-informed perturbations and consensus clustering
Source: PLoS Comput Biol. 2026 Jun 18;22(6):e1014167. doi: 10.1371/journal.pcbi.1014167 (PMC13293510; doi:10.1371/journal.pcbi.1014167)
Supplement: S1 Text — (PDF) [file pcbi.1014167.s014.pdf]

Supplementary Materials for

scMagnifier: resolving fine-grained cell subtypes via GRN-informed perturbations and consensus clustering

Zhenhui He[1] and Kangning Dong[1*]

1School of Mathematics, Renmin University of China, Beijing 100872, China
*Correspondence: dongkangning@ruc.edu.cn.

Supplementary Tabels

Table A Description of all scRNA-seq datasets used in this study.
Table B Description of ST datasets used in this study.
Table C Benchmarking results of scMagnifier on single-batch datasets based on ARI.
Table D Benchmarking results of scMagnifier on single-batch datasets based on NMI.
Table E Benchmarking results of scMagnifier on single-batch datasets based on Silhouette Score.
Table F Benchmarking results of scMagnifier on single-batch datasets based on Entropy of cell type mixing.
Table G Benchmarking results of scMagnifier on multi-batch datasets based on ARI.
Table H Benchmarking results of scMagnifier on multi-batch datasets based on NMI.
Table I Benchmarking results of scMagnifier on multi-batch datasets based on Silhouette Score.
Table J Benchmarking results of scMagnifier on multi-batch datasets based on Entropy of batch mixing.
Table K Ablation study results of scMagnifier on ARI.
Table L Ablation study results of scMagnifier on NMI.
Table M Ablation study results of scMagnifier on Silhouette Score.
Table N Sensitivity analysis of scMagnifier perturbation fold change on the EBUS_10 dataset.
Table O Sensitivity analysis of scMagnifier perturbation fold change on the LUNG_N09 dataset.
Table P Sensitivity analysis of scMagnifier perturbation fold change on the LUNG_N34 dataset.
Table Q Jaccard similarity coefficients between the sets of the differentially expressed genes for R1/R2 and each real cell type in the EBUS_10 dataset.
Table R Jaccard similarity coefficients between the sets of the differentially expressed genes for R3 and each real cell type in the LUNG_N30 dataset.
Table S Number of perturbed TFs for each dataset.

Table A Description of all scRNA-seq datasets used in this study.

Dataset	Cells	Genes	Types	Accession	Description
LN_04	2584	29634	8	GSE131907	Lung adenocarcinoma cells
EBUS_10	4477	29634	15	GSE131907	Lung adenocarcinoma cells
LUNG_N09	2022	29634	12	GSE131907	Lung adenocarcinoma cells
LUNG_N30	2886	29634	16	GSE131907	Lung adenocarcinoma cells
LUNG_N34	4079	29634	14	GSE131907	Lung adenocarcinoma cells
Pancreas	8569	20125	14	GSE84133	Human pancreas cells
UPN19_pre	10890	19957	14	huARdb	T-cell type large granular lymphocyte leukemia cells
BMMC	69249	13431	22	GSE194122	Human bone marrow mononuclear cells

Table B Description of ST datasets used in this study.

Dataset	Spots	Genes	Platform	Accession	Description
OV	48793	5001	Xenium 5K	SPATCH	ovarian cancer cells

Table C Benchmarking results of scMagnifier on single-batch datasets based on ARI.

	Leiden	Louvain	scVI(Leiden)	scVI(Lovain)	SC3s	DBSCAN	Hierarchical	scMagnifier (Leiden)	scMagnifie (Louvain)
LN_04	0.3599	0.4181	0.3682	0.4454	0.3504	0.4203	0.3969	0.5983	0.6378
EBUS_10	0.6472	0.4693	0.4329	0.4200	0.6274	0.3755	0.8289	0.8180	0.8033
LUNG_N09	0.5845	0.5135	0.5753	0.5118	0.6287	0.6501	0.9098	0.8686	0.8933
LUNG_N34	0.7740	0.7392	0.7470	0.7132	0.6965	0.5833	0.8683	0.8711	0.8692

Table D Benchmarking results of scMagnifier on single-batch datasets based on NMI.

	Leiden	Louvain	scVI(Leiden)	scVI(Lovain)	SC3s	DBSCAN	Hierarchical	scMagnifier (Leiden)	scMagnif (Louvai
LN_04	0.5300	0.5976	0.5690	0.6026	0.4871	0.4346	0.5371	0.6075	0.6223
EBUS_10	0.7774	0.7395	0.6984	0.6896	0.7355	0.4981	0.7862	0.8073	0.7992
LUNG_N09	0.7804	0.7702	0.7889	0.7752	0.8162	0.6319	0.8792	0.8513	0.8707
LUNG_N34	0.8556	0.8450	0.8259	0.8173	0.8305	0.6962	0.8686	0.8532	0.8567

Table E Benchmarking results of scMagnifier on single-batch datasets based on Silhouette Score.

	Leiden	Louvain	scVI(Leiden)	scVI(Lovain)	SC3s	DBSCAN	Hierarchical	scMagnifier (Leiden)	scMagnifi (Louvain
LN_04	0.1853	0.1380	0.1644	0.1914	0.4687	0.0721	0.2052	0.2824	0.2469
EBUS_10	0.2501	0.2105	0.1443	0.1360	0.4460	0.1223	0.3321	0.4883	0.4335
LUNG_N09	0.3028	0.2758	0.3050	0.2838	0.4845	0.3205	0.5608	0.4162	0.4834
LUNG_N34	0.3882	0.3670	0.2745	0.2643	0.6173	0.3445	0.4688	0.5251	0.4673

Table F Benchmarking results of scMagnifier on single-batch datasets based on Entropy of mixing.

	scMagnifier	PCA	scVI
LUNG_N09	0.0776	0.0873	0.0942
LUNG_N34	0.0910	0.1018	0.1306
EBUS_10	0.1172	0.1355	0.2246
LN_04	0.1564	0.4399	0.1756

Table G Benchmarking results of scMagnifier on multi-batch datasets based on ARI.

	BBKNN	Harmony	scMagnifier+Harmony	Scanorama	scMagnifier+Scnorama	scVI	scMagnifier+scVI
Pancreas	0.6193	0.6759	0.8620	0.7089	0.8327	0.8540	0.9273
BMMC	0.5410	0.5472	0.6082	0.5870	0.5860	0.6170	0.6581

Table H Benchmarking results of scMagnifier on multi-batch datasets based on NMI.

	BBKNN	Harmony	scMagnifier+Harmony	Scanorama	scMagnifier+Scnorama	scVI	scMagnifier+scVI
Pancreas	0.7978	0.8283	0.8509	0.8396	0.8752	0.8688	0.9047
BMMC	0.6990	0.7105	0.7255	0.7372	0.7457	0.7376	0.7418

Table I Benchmarking results of scMagnifier on multi-batch datasets based on Silhouette Score.

	BBKNN	Harmony	scMagnifier+Harmony	Scanorama	scMagnifier+Scnorama	scVI	scMagnifier+scVI
Pancreas	0.2824	0.2798	0.3356	0.1610	0.2809	0.1959	0.3713
BMMC	0.1935	0.2061	0.4964	0.1218	0.3102	0.1143	0.2340

Table J Benchmarking results of scMagnifier on multi-batch datasets based on Entropy of batch mixing.

	BBKNN	Harmony	scMagnifier+Harmony	Scanorama	scMagnifier+Scnorama	scVI	scMagnifier+scVI
Pancreas	0.1844	0.6035	0.6001	0.4756	0.4577	0.4590	0.4449
BMMC	0.3687	0.5808	0.5793	0.5211	0.5046	0.5687	0.5328

Table K Ablation study results of scMagnifier on ARI.

	Remove cluster merge	Remove GEM-derived distance	Remove perturbation-derived distacne	Full scMagnifier
LN_04	0.4255	0.6531	0.3899	0.6313
EBUS_10	0.6019	0.8140	0.8030	0.8247
LUNG_N09	0.5700	0.5049	0.8732	0.9004

Table L Ablation study results of scMagnifier on NMI.

	Remove cluster merge	Remove GEM-derived distance	Remove perturbation-derived distance	Full scMagnifier
LN_04	0.6099	0.6251	0.5623	0.6200
EBUS_10	0.7658	0.8078	0.7798	0.8118
LUNG_N09	0.7914	0.7403	0.8662	0.8710

Table M Ablation study results of scMagnifier on Silhouette Score.

	Remove cluster merge	Remove GEM-derived relationships	Remove perturbation-derived relationships	Full scMagnifier
LN_04	0.5380	0.4081	0.1190	0.2623
EBUS_10	0.5547	0.6552	0.3447	0.4950
LUNG_N09	0.5565	0.4820	0.4374	0.4840

Table N Sensitivity analysis of scMagnifier perturbation fold change on the EBUS_10 dataset.

	±5%	±10%	±20%	±30%	±40%	±50%
ARI	0.8141	0.8172	0.8141	0.7978	0.8047	0.7940
NMI	0.8188	0.8162	0.8189	0.8014	0.8096	0.8084
SIL	0.4716	0.4954	0.4702	0.4646	0.4675	0.4686

Table O Sensitivity analysis of scMagnifier perturbation fold change on the LUNG_N09 dataset.

	±5%	±10%	±20%	±30%	±40%	±50%
ARI	0.7710	0.7917	0.7547	0.7204	0.6930	0.6608
NMI	0.7655	0.7920	0.7640	0.7544	0.7487	0.7541
SIL	0.2236	0.2300	0.2199	0.1935	0.2024	0.2023

Table P Sensitivity analysis of scMagnifier perturbation fold change on the LUNG_N34 dataset.

	±5%	±10%	±20%	±30%	±40%	±50%
ARI	0.8718	0.8730	0.8747	0.8747	0.8748	0.8748
NMI	0.8549	0.8544	0.8582	0.8582	0.8582	0.8582
SIL	0.5216	0.5289	0.5212	0.5157	0.5159	0.5146

Table Q Jaccard similarity coefficients between the sets of the differentially expressed genes for R1/R2 and each real cell type in the EBUS_10 dataset.

	R1	R2
Follicular B cells	0.0204	0.0101
Malignant cells	0.0000	0.0000
CD163+CD14+ DCs	0.0101	0.0417
mo-Mac	0.0000	0.0101
GC B cells in the DZ	0.3333	0.4085
Naive CD8+ T	0.0204	0.0000
GC B cells in the LZ	0.0638	0.1364
Naive CD4+ T	0.0000	0.0000
Undetermined	0.0417	0.1111
Monocytes	0.0000	0.0101
Treg	0.0204	0.0417
MALT B cells	0.1236	0.0101
Exhausted CD8+ T	0.0309	0.0753
CD8 low T	0.0204	0.0417
NK	0.0000	0.0000

Table R Jaccard similarity coefficients between the sets of the differentially expressed genes for R3 and each real cell type in the LUNG_N30 dataset.

	R3
Ciliated	0.0000
NK	0.3699
AT2	0.0000
CD8 low T	0.2048
Cytotoxic CD8+ T	0.1905
Club	0.0000
CD4+ Th	0.0309
Monocytes	0.0204
Lymphatic ECs	0.0101
COL14A1+ matrix FBs	0.0000
Alveolar Mac	0.0000
mo-Mac	0.0101
MAST	0.0417
COL13A1+ matrix FBs	0.0000
Tip-like ECs	0.0526
CD1c+ DCs	0.0000

Table S Number of perturbed TFs for each dataset.

	LN_04	EBUS_10	LUNG_N09	LUNG_N30	LUNG_N34	Pancreas	UPN19_pre	BMMC	OV
TFs Number	102	82	82	87	80	149	110	67	68
